# Cyanobacterial exopolymer properties differentiate microbial carbonate fabrics

**DOI:** 10.1038/s41598-017-12303-9

**Published:** 2017-09-18

**Authors:** Fumito Shiraishi, Yusaku Hanzawa, Tomoyo Okumura, Naotaka Tomioka, Yu Kodama, Hiroki Suga, Yoshio Takahashi, Akihiro Kano

**Affiliations:** 1 Department of Earth and Planetary Systems Science, Graduate School of Science, Hiroshima University, Hiroshima, 739-8526 Japan; 2 Department of Subsurface Geobiological Analysis and Research (D-SUGAR), Japan Agency for Marine-Earth Science and Technology (JAMSTEC), Yokosuka, 237-0061 Japan; 3 Department of Earth and Planetary Science, The University of Tokyo, Tokyo, 113-0033 Japan; 4 Kochi Institute for Core Sample Research, JAMSTEC, Kochi, 783-8502 Japan; 5 Marine Works Japan Ltd., Yokosuka, 237-0063 Japan

## Abstract

Although environmental changes and evolution of life are potentially recorded via microbial carbonates, including laminated stromatolites and clotted thrombolites, factors controlling their fabric are still a matter of controversy. Herein, we report that the exopolymer properties of different cyanobacterial taxa primarily control the microbial carbonates fabrics in modern examples. This study shows that the calcite encrustation of filamentous *Phormidium* sp. secreting acidic exopolymers forms the laminated fabric of stromatolites, whereas the encrustation of coccoid *Coelosphaeriopsis* sp. secreting acidic exopolymers and poor calcification of filamentous *Leptolyngbya* sp. secreting non-acidic exopolymers form peloids and fenestral structures, respectively, *i.e*. the clotted fabric of thrombolites. Based on these findings, we suggest that the rise and decline of cyanobacteria possessing different exopolymer properties caused the expansion of thrombolites around the Proterozoic/Cambrian boundary.

## Introduction

The formation of organosedimentary deposits known as microbialites by benthic microbial communities began perhaps as early as 3.7 billion years ago^1^ and led to substantial amounts of carbonate deposits in the rock record thereafter^2,3^. Microbialites are classified according to their internal fabrics at the mesoscopic scale (millimetre to centimetre scale), where stromatolites (laminated fabric) and thrombolites (clotted fabric) are the dominant types^4,5^. However, the biogeochemical processes that control their fabrics are still a matter of controversy. Microbial carbonates were common in ancient marine environments; however, they are currently restricted to terrestrial and littoral settings possibly due to changes in seawater chemistry^6^. An example of modern microbial carbonates is identified in tufa, which is a carbonate deposit that develops along karst creeks and in lakes^7^, in which cyanobacterial photosynthesis frequently induces calcite precipitation to form stromatolites^8,9^. We have recently discovered a unique tufa site in Japan with deposits of not only stromatolites (tufa stromatolites^5^) but also thrombolites (tufa thrombolites^5^), which provides an exceptional opportunity to examine the key factors affecting microbial carbonate fabrics because the development of two microbialites at the same site significantly reduces the number of factors in need of consideration^10^.

## Results

### Field settings

The Ueno tufa site (34°54′N, 133°33′E) is located on the Atetsu Limestone Plateau in southwestern Japan (Supplementary Fig. S1a,b). Here groundwater discharges from a limestone fracture and forms a small creek that is approximately 30-m long. The upper and lower reaches of the stream are dominated by moss tufa and thrombolite, respectively (Supplementary Fig. S1c,d). Stromatolite was developed along the eastern flow path of the lower reach. Potential grazers (e.g. protozoa, nematodes and gastropods) are scarce in this site.

### Major mineralogy and water chemistry

Powder X-ray diffraction (XRD) patterns show that the major mineralogy for both the stromatolites and thrombolites is calcite (Supplementary Fig. S1e,f). As we move downstream, decreases in the Ca^2+^ concentration and alkalinity of the creek water coupled with increases in the pH and calcite saturation state (Ω) (Supplementary Fig. S1g), which are common features of general tufa-depositing creeks, reflect progressive CO_2_ degassing and calcite precipitation.

### Bacterial composition

The composition of the bacterial community estimated by 16 S rRNA gene clone library analysis shows that the phylum Cyanobacteria is dominant in both the stromatolites and thrombolites (Supplementary Fig. S2). However, the bacterial diversity in the stromatolites is lower than that in the thrombolites possibly due to the higher flow velocity at the stromatolite surface: it is experimentally demonstrated that the bacterial diversity in freshwater biofilms inversely correlates with the flow velocity^11^. This hydrodynamic effect would also differentiate the dominant cyanobacterial genera: genus *Phormidium* is dominant in the stromatolites, whereas genus *Leptolyngbya* is dominant in the thrombolites. This trend of cyanobacterial phylotype composition is consistent with that of morphotypes identified by the microscopic observations described below.

### Metabolic influence on CaCO_3_ precipitation

Microelectrode measurements show similar trends in both the stromatolites and thrombolites (Supplementary Fig. S3). Under light conditions, increases of O_2_, CO_3_^2−^ and Ω and decreases of CO_2_ and Ca^2+^ are observed at the microbialite surface. Under dark conditions, a decrease of O_2_ and an increase of CO_2_ are observed, whereas Ca^2+^, CO_3_^2−^ and Ω exhibit no detectable shift. These results indicate that CaCO_3_ precipitation at both microbialites is primarily induced by cyanobacterial photosynthesis^9^.

### Depositional and mineralogical characteristics of stromatolite

**Figure 1:**
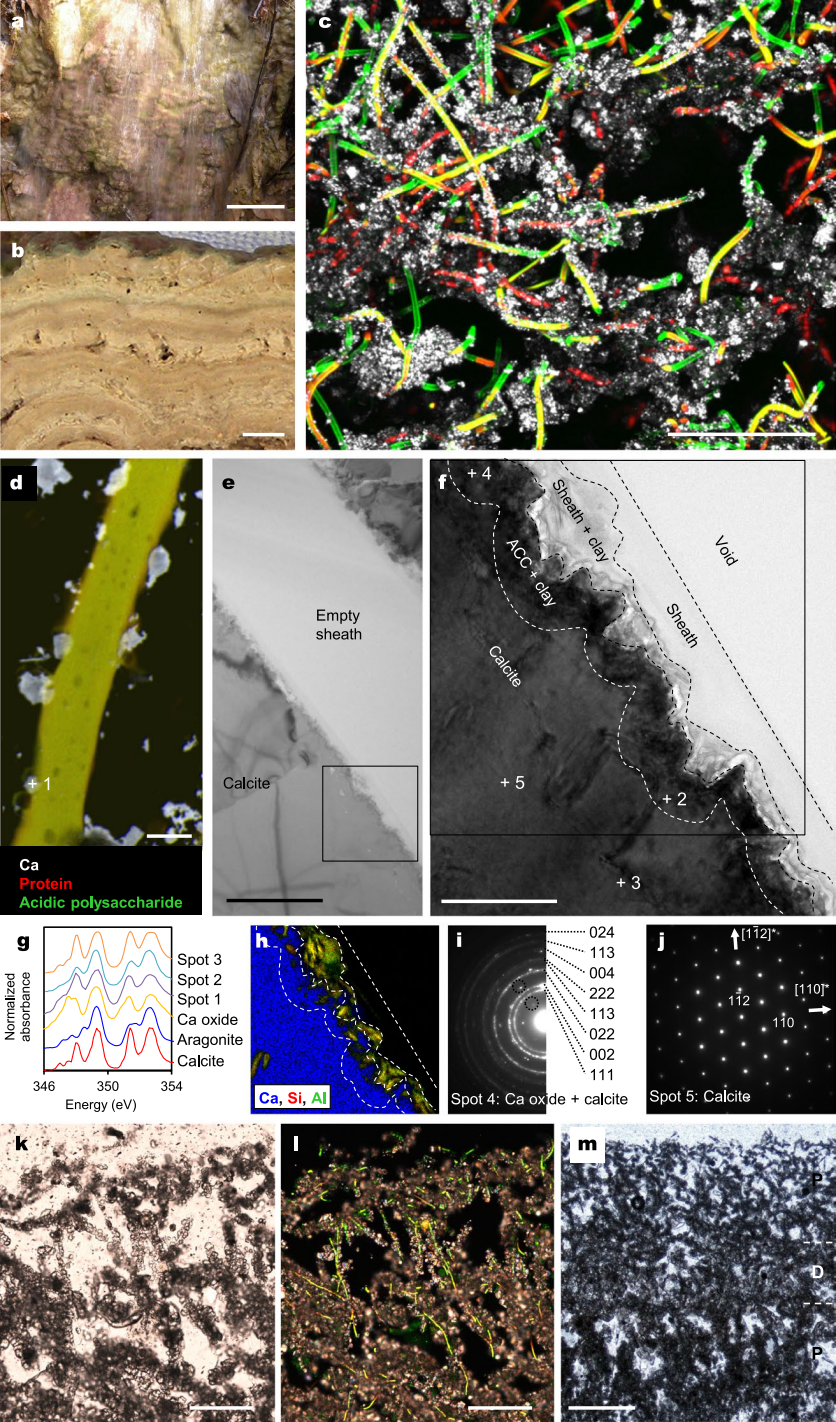
Depositional and mineralogical characteristics of stromatolite. (**a**) Close-up view of stromatolite surface in the field, upon which water flows relatively fast. (**b**) Cross-section of stromatolite surface. (**c**) CLSM image of stromatolite surface with LBA staining. Acidic exopolymers (green fluorescence) of *Phormidium* sp. (yellow-red autofluorescence) are surrounded by minerals (white reflected light). (**d**) STXM-based compositional image of mineralized *Phormidium* sp. NEXAFS analysis spot is indicated (spot 1). (**e**) Bright-field TEM image showing a mineralized empty sheath of *Phormidium*. (**f**) Magnified region from (**e**). NEXAFS analysis spots (spots 2 and 3) and selected area electron diffraction (SAED) patterns (spots 4 and 5) are indicated. (**g**) Ca 2p NEXAFS spectra. (**h**) Combined elemental map of the region indicated in (**f**). (**i**) SAED pattern taken from spot 4. The numbers are the indices of diffraction rings from a polycrystalline aggregate of calcium oxide. Dashed circles indicate examples of overlapped diffraction spots from a calcite crystal. (**j**) SAED pattern of a calcite crystal taken from spot 5. (**k**,**l**) Thin section images of stromatolite surfaces showing the same microscopic field of view; (**k**) transmitted light image and (**l**) a composite of cross-polarized and fluorescence light images. *Phormidium* filaments are recognized as yellow autofluorescence in (**l**). (**m**) Transmitted light image, as in (**k**), but showing a larger area. Porous (P) and dense (D) layers were indicated. Scale bars: (**a**) 5 cm; (**b**) 1 cm; (**c**,**k**,**l**) 100 μm; (**d**,**e**) 2 μm; (**f**) 500 nm; (**m**) 500  μm.

The surface of stromatolites is represented by patches of light green and purple colour, and several millimetres think laminations are recognised in the cross section (Fig. 1a, b). Confocal laser scanning microscopy (CLSM) and transmission electron microscopy (TEM) observations indicate that the dominant cyanobacterium in stromatolites is a filamentous *Phormidium* sp. that secretes a thin (ca. 0.2 μm) exopolymer sheath (Fig. 1c; Supplementary Fig. S4a). Lectin binding analysis (LBA) suggests that these sheaths contain abundant acidic sugars with carboxyl groups and the sheath exterior is surrounded by a significant number of fine minerals (Fig. 1c). TEM and scanning transmission X-ray microscopy (STXM) observations reveal that the minerals larger than ca. 1 μm in diameter are mostly calcite (Fig. 1d–j). Magnesium is undetectable in these calcite crystals (Supplementary Fig. S5), which is consistent with the feature of carbonates precipitated around the exopolymers^12^. In the vicinity of the exopolymer sheath, minerals exhibit the characteristics of an amorphous CaCO_3_ (ACC) precursor reported by ref.^13^: they show aragonite-like NEXAFS (near edge X-ray absorption fine structure) spectra (Fig. 1f,g), and are unstable under an electron beam, and decompose into polycrystalline calcium oxide during TEM observations (Fig. 1i). These features suggest that calcite nucleation occurred on the surface of a *Phormidium* sheath. Conversely, minerals that are ca. 200 nm in diameter are mostly clay minerals (Fig. 1f,h; Supplementary Fig. S4a) representing the absorption of suspended clay from turbulent water onto the acidic exopolymer via a divalent cation bridge^14^. Heavy calcification of *Phormidium* filaments results in sheath encrustation^15^, and abundant, upward oriented filaments at the stromatolite surface (Fig. 1k,l) cause horizontally uniform precipitation of calcite at the mesoscopic scale. The spaces between calcified filaments are left as growth cavities, and the alternation of porous and dense layers forms laminations (Fig. 1m).

### Depositional and mineralogical characteristics of thrombolite

**Figure 2:**
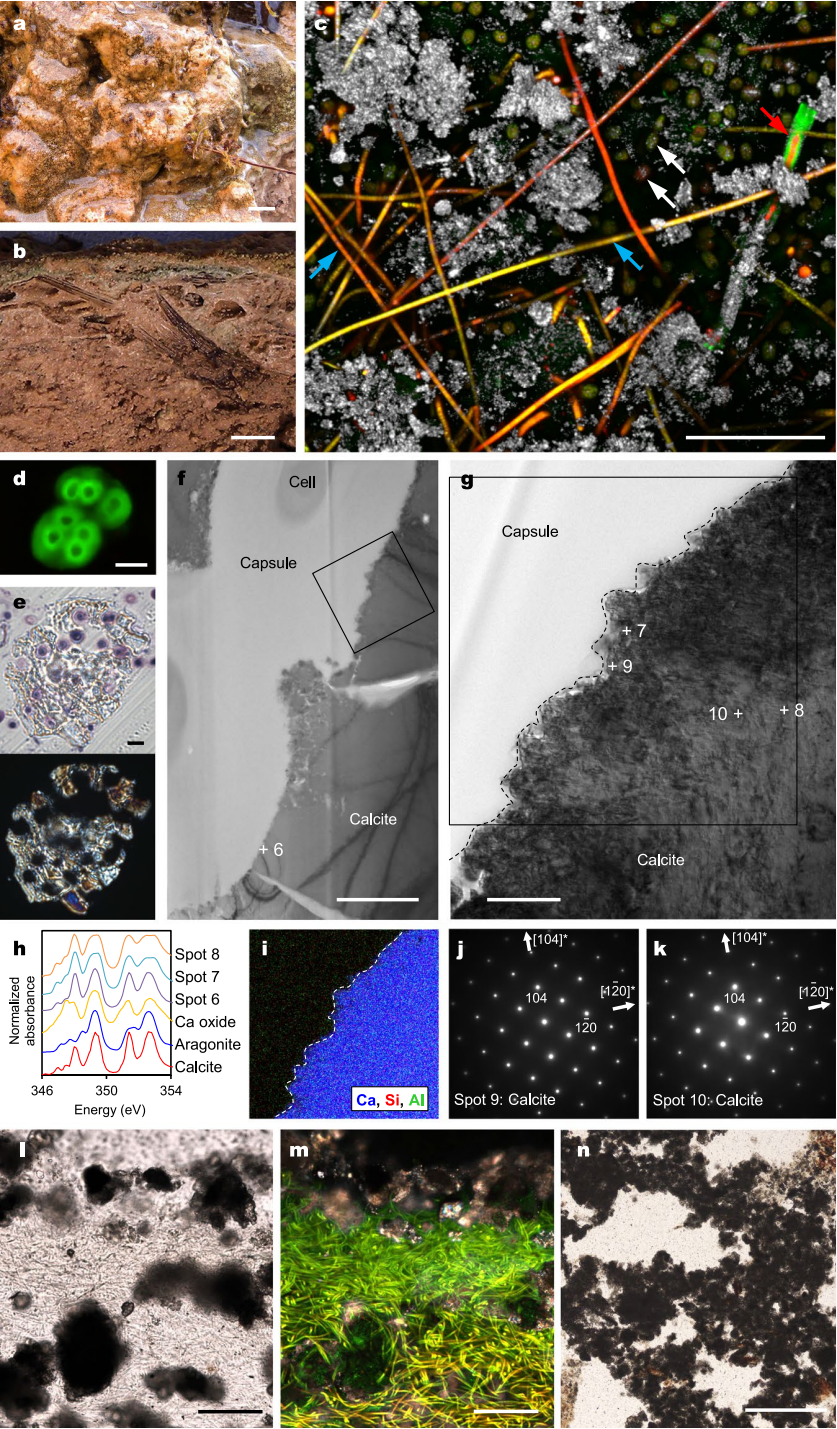
Depositional and mineralogical characteristics of thrombolite. (**a**) Close-up view of thrombolite surface in the field, covered by a thin film of water. (**b**) Cross-section of thrombolite surface. (**c**) CLSM image of thrombolite surface with LBA staining. Observed phototrophs (yellow-red autofluorescence) are *Leptolyngbya* sp. (e.g., cyan arrows), *Scytonema* sp. (red arrow), and unicellular eukaryotic microalga *Oocardium* sp. (e.g., white arrows). Minerals (white reflected light) are present as peloids and fillings of acidic exopolymers (green fluorescence) secreted by *Scytonema* sp. (**d**) CLSM image of *Coelosphaeriopsis* sp. with LBA staining. (**e**) Transmitted (top) and cross-polarized (bottom) light images of a single peloid grain acquired from an 800 nm thin section. Rounded spaces inside of peloids are occupied by *Coelosphaeriopsis* sp. (**f**) Bright-field TEM image showing mineralized capsules of *Coelosphaeriopsis* sp. The NEXAFS analysis spot is indicated (spot 6). (**g**) Magnified region from (**f**). NEXAFS analysis spots (spots 7 and 8) and SAED patterns (spots 9 and 10) are indicated. (**h**) Ca 2p NEXAFS spectra. (**i**) Combined elemental map of the region indicated in (**g**). (**j**,**k**) SAED patterns of calcite crystals taken from (**j**) spots 9 and (**k**) 10. (**l**,**m**) Thin section images of thrombolite surfaces with the same microscopic field of view; (**l**) transmitted light image and (**m**) a composite of cross-polarized and fluorescence light images. *Leptolyngbya* filaments are recognized by yellow-green autofluorescence in (**m**). (**n**) Transmitted light image, as in (**l**), but showing the deeper part. Scale bars: (**a**,**b**) 1 cm; (**c**, **l**, **m**) 100 μm; (**d**,**e**) 5 μm; (**f**) 2 μm; (**g**) 500 nm; (**n**) 500 μm.

The surface of thrombolites is orange, and no laminations are observed in its cross section (Fig. 2a,b); instead, the submillimetre to 1-cm-sized fenestral structures are conspicuous (Fig. 2n). CLSM and TEM observations indicate that the dominant cyanobacteria are filamentous *Leptolyngbya* sp. and coccoid *Coelosphaeriopsis* sp., which secrete a thin (ca. 0.3−0.5 μm) exopolymer sheath and a relatively thick (ca. 1.5−3.0 μm) capsule, respectively (Fig. 2c,d; Supplementary Fig. S4b,c). LBA suggests that *Leptolyngbya* sheaths lacking a detectable amount of acidic sugars are mostly free from mineralisation (Fig. 2c), whereas *Coelosphaeriopsis* capsules containing acidic sugars are enclosed by minerals (Fig. 2d,e). TEM and STXM observations reveal that calcite is the encrusting mineral on *Coelosphaeriopsis* capsules (Fig. 2f–k). In a conventional thin section, calcified *Coelosphaeriopsis* colonies exhibit peloids, which are internally structureless microcrystalline carbonate sands^16^ (Fig. 2l–n). The peloids are scattered around tangled *Leptolyngbya* filaments at the thrombolite surface (Fig. 2l,m). They become more condensed in the deeper parts of thrombolites, and the spaces occupied by non-mineralised *Leptolyngbya* filaments are left as irregular fenestral structures, with overall formation of clotted fabrics at the mesoscopic scale (Fig. 2n). In addition, a filamentous cyanobacterium *Scytonema* sp. that secretes a relatively thick (ca. 3–5 μm) and acidic exopolymer sheath is locally visible in the thrombolite, and the sheath interior is impregnated with calcite crystals (Fig. 2c)^15,17^.

The acidic sugar detected by LBA was confirmed by a lectin blocking assay (Supplementary Fig. S6) and the fluorescence labelling of carboxyl groups (Supplementary Fig. S7).

## Discussion

### Factors controlling the microbial carbonate fabrics

Among the observations at the Ueno tufa site, the spatial proximity of heavily and poorly calcified cyanobacteria that secret acidic and non-acidic exopolymers, respectively, in the thrombolites is critical toward understanding the microbial carbonate formation mechanism. The crystallisation process generally comprises crystal nucleation and growth, and both the nucleation and growth rates are proportional to mineral supersaturation^18,19^. Supersaturation at the microbialite surface, which is the major crystallisation site, is elevated by the combination of CO_2_ degassing in the water column and cyanobacterial photosynthesis at the deposit surface (Supplementary Fig. S3). However, a diffusive boundary layer (DBL) blankets the deposit surface^20^, and a significant difference in supersaturation cannot be expected at the mesoscopic scale. In contract, supersaturation of water retained in the exopolymer sheaths/capsules is likely much higher under light conditions due to a reduced diffusion rate^21^. Very high supersaturation in the exopolymer sheaths/capsules *per se* is common to most cyanobacterial taxa; however, the calcite nucleation rate on polysaccharides is proportional to their acidity under such conditions^22^. Therefore, the observed difference in the degree of cyanobacterial calcification can be primarily attributed to the difference in their exopolymer acidity, *i.e*. the nucleation rate. This interpretation also applies to stromatolites, although cation absorption by clay minerals may affect the formation and stability of the ACC precursor. Higher flow velocity at the stromatolite-depositing site would reduce the DBL thickness^23^; however, it reduces the hydrochemical difference between the stromatolite surface and the water column, which cannot create a significant difference of supersaturation at the mesoscopic scale. The calcification styles of acidic exopolymer sheaths/capsules, either as encrustation or impregnation, are not apparently related to their thickness, which indicates the influence of other factors such as their internal structure.

The cyanobacterial exopolymer properties described so far are further responsible for the differentiation of the fabrics of the investigated microbialites. The dominance of filamentous cyanobacteria that secrete acidic exopolymers provides mesoscopically uniform nucleation sites, owing to which the laminated fabric of stromatolites is produced. In contrast, a combination of coccoid and filamentous cyanobacteria that secrete acidic and non-acidic exopolymers, respectively, provides mesoscopically heterogeneous nucleation sites, owing to which the clotted fabric of thrombolites is formed. These observations indicate that three-dimensional structures of biofilms strongly affect microbialite fabrics. The cyanobacterial cellular morphology, either coccoid or filamentous, has a subordinate influence by affecting the distribution pattern of nucleation sites. In addition, small coccoid cyanobacteria observed inside peloids (Fig. 2e) are hardly recognisable in a conventional thin section (ca. 50-μm thick; Fig. 2l–n), which potentially resolves the controversy regarding the relation between dominant cellular morphology and microbialite fabrics^2,4,10^.

These results have improved our knowledge of the fundamental mechanisms involved in the formation of microbial carbonates, as follows:

1. Confirmation of the long-held view that cyanobacterial acidic exopolymers provide CaCO_3_ mineral nucleation sites^15^.
2. Recognition that non-acidic exopolymers are relatively unsuitable for nucleation, which overwhelms the inhibitory effect of acidic exopolymers^21,24^.
3. Supersaturation at crystallisation sites primarily contributes to the precipitation quantity rather than localizes the nucleation sites, which suggests that it is a prerequisite for microbial carbonate formation, as previously assumed^5,21,24^.

### Implications for the fossil record

These observations from modern examples provide significant insights into the interpretation of the fossil records of microbial carbonate, particularly from the perspective of their fabric and quantity. For example, a substantial change of microbial carbonate fabric occurred around the Proterozoic/Cambrian boundary when both thrombolites and calcified cyanobacteria first expanded^4–6,15,25^. A number of factors have been proposed to explain these changes: those for thrombolites include dominant microbial cellular morphology^4^, a framework construction mechanism^2^ and a microbial growth/calcification ratio^10^, whereas those for calcified cyanobacteria include the ambient water Mg^2+^/Ca^2+^ ratio^26^, temperature^27^, CaCO_3_ supersaturation^28^, dissolved inorganic carbon (DIC) concentration^21^ and equilibrium CO_2_ partial pressure (*p*CO_2_)^29^. However, our observations from modern processes underscore the potential importance of cyanobacterial exopolymer properties to the expansions of both of thrombolites and calcified cyanobacteria around the Proterozoic/Cambrian boundary. If this is the case, an evolutionary/extinction event of cyanobacteria that drastically changed their exopolymer properties is expected at that time.

Conversely, long-term quantitative changes recognised in the fossil record^3,21^ would have been largely, if not entirely, controlled by supersaturation at the crystallisation sites. Indeed, this view has been experimentally corroborated^9^. By evaluating factors other than supersaturation (such as precipitation inhibitors^26^ and metazoan competition^3^), the quantitative records of microbial carbonate would provide a proxy for oceanic pH and DIC^9^.

These interpretations echo the perceptive view presented in ref.^6^ that the history of microbial carbonates reflects the superimposition of prokaryote evolutionary/extinction events onto environmental fluctuations. In any case, future studies must evaluate the applicability of knowledge from freshwater microbialites to their seawater counterparts.

## Methods

### XRD analysis

The surface part (ca. 5 mm) of microbialite samples were air-dried, powdered using a mortar and pestle, and analyzed using a powder X-ray diffractometer with Cu Kα radiation (40 kV, 40 mA) and a graphite monochromator (MultiFlex, Rigaku).

### Water chemistry analysis

For characterization of the creek water chemistry, pH and temperature were measured in the field using a portable pH meter (D-51, Horiba). Alkalinity was determined by acid-base titration using a hand-held titrator and a 1.6 N H_2_SO_4_ cartridge (Hach). Water samples filtered through a 0.2 μm membrane (Minisart, Sartorius) were collected in plastic bottles, and anion concentrations (Cl^−^, NO_3_^−^, and SO_4_^2−^) were measured using ion chromatography (ICS-1100, Thermo Fisher Scientific). Aliquots of filtrated samples were adjusted to 2% HNO_3_, and cation concentrations (Ca^2+^, Mg^2+^, Na^+^, and K^+^) were estimated using inductively coupled plasma optical emission spectroscopy (ICP-OES; iCAP7200, Thermo Fisher Scientific). The measured values were processed with the PHREEQC^30^ computer program to calculate the DIC concentration, *p*CO_2_, and Ω.

### Carbon and oxygen stable isotope analysis

Water samples were filtered through a 0.2 μm membrane and collected in gas-tight glass bottles. Tufa samples were collected by scraping the surface part (ca. 0.5 mm) with a knife, and air-drying. Carbon and oxygen isotopes were measured with a mass spectrometer, as described previously^31^.

### **1**6 S rRNA gene analysis

Almost full-length 16 S rRNA genes of bacteria were obtained from stromatolite and thrombolite, for which the methods described previously^32^ were applied for sampling, DNA extraction, and polymerase chain reaction (PCR) amplification. The PCR products were purified, cloned into vector pTAC-1 (BioDynamics Laboratory Inc.), and then transformed into chemically competent *Escherichia coli* (Competent Quick DH5α, Toyobo). Inserts of randomly selected colonies were used for bidirectional sequencing with flanking vector primer M13BDFw (5′ CAG GGT TTT CCC AGT CAC GAC 3′) and M13BDRev (5′ CGG ATA ACA ATT TCA CAC AGG 3′). DNA sequencing was performed on a DNA analyzer (ABI3730, Applied Biosystems) with the BigDye terminator version 3.1 cycle sequencing kit. Closest relatives were determined using SINA online 16 S rRNA sequence classifier^33^ based on the Greengens database^34^. The obtained sequences (76 clones from the stromatolite, and 99 clones from the thrombolite) were checked for their chimeras using the Bellerophon server^35^. Representative gene sequences in this study were deposited in the DNA Data Bank of Japan (DDBJ) database under accession numbers AB862884–AB862938, and LC215056–LC215137.

### Microelectrode measurements

Microbial metabolism and CaCO_3_ precipitation at the microbialite surface was evaluated using O_2_, CO_2_, Ca^2+^, and CO_3_^2−^ microelectrodes, as described previously^9^. Construction and handling of the CO_3_^2−^ microelectrode were performed according to ref.^36^. Creek water collected at Site 1 was used for the measurement.

### Thin section observations

Vertical sections of the microbialite surfaces were observed using thin sections. Microbialite samples were first fixed using phosphate-buffered saline (PBS) containing 3.7% formaldehyde for 2 days, after which the solution was replaced with 50% ethanol in PBS and the sample was stored at 4 °C until further processing. Thin sections were then prepared from resin-embedded samples, as described previously^37^. Transmitted, cross-polarized, and fluorescent light images were acquired using CLSM (LSM700, Zeiss) equipped with a CCD camera (AxioCam MRc, Zeiss) and ZEN2010 software (Zeiss). Fluorescent light images consisted of two channels, one acquired by excitation at 488 nm with a BP505-600 nm emission filter, and another by excitation at 555 nm with an LP615 nm emission filter. Composites of cross-polarized and fluorescent light images were generated by the Lighten mode of Adobe Photoshop CS6. Transmitted light images of lower magnification were acquired using a conventional microscope (Eclipse LV100 POL, Nikon).

### LBA

The distribution pattern of acidic sugars was investigated using a lectin from *Limulus polyphemus* (LPA, Cosmo Bio), which is known to have binding specificity to glucuronic acid and N-acetylneuraminic acid^38^. Either small blocks of the surface part (ca. 5 mm^3^) or a slurry were prepared from fresh microbialite samples (within 48 h after collection), and soaked in 50 ng μL^−1^ of FITC-conjugated LPA lectin for 20 min at room temperature. Unbound lectin was thoroughly removed by washing with a buffer [88 mM NaCl, 20 mM Tris (pH 8.0), 0.01% (w/v) SDS], and block samples were submerged in distilled water while slurry samples were enclosed by a cover slip with mounting media (AF2, Citifluor). Fluorescent and reflected light (488 nm excitation with an SP490 nm emission filter) images were then acquired using CLSM. For block samples, image stacks of optical slices were acquired, and plane views of the rough microbialite surface were generated by maximum intensity projection mode of Imaris software (Bitplane). Negative control of LBA was conducted by applying FITC-conjugated lectin from *Phaseolus vulgaris* (PHA, Cosmo Bio), which has binding specificity to none of the tested sugars^38^. In addition, untreated samples were observed for comparison.

### Lectin blocking assay

Binding specificity of LPA lectin was checked by lectin blocking assay^38^. 12 competing sugars were tested: 11 were selected from 12 different monosaccharides identified from cyanobacterial exopolymers to date (glucuronic acid, galacturonic acid, arabinose, fructose, fucose, galactose, glucose, mannose, rhamnose, ribose, and xylose)^39^, and 1 was N-acetylneuraminic acid. First, 50 ng μL^−1^ of FITC-conjugated LPA lectin was pre-incubated for 15 min with competing sugars at three different concentrations (0.1, 1, and 10 mg mL^−1^), followed by LBA using stromatolite samples as described above. The same microscopic settings were applied for all samples.

### Fluorescence labeling of carboxyl groups

To cross-check the results of the LBA, carboxyl groups were fluorescently labeled by modification of the procedure described previously in ref.^40^. Either block or slurry samples fixed with 3.7% formaldehyde/PBS were soaked in 2 mL of 0.1 M 2-morpholinoethanesulfonic acid (MES) buffer, pH 5.5, and 100 μL of 50 mM EZ-Link Pentylamine-Biotin (Thermo Fisher Scientific) was added, followed by 25 μL of 100 mg mL^−1^ 1-ethyl-3-(3-dimethylaminopropyl)carbodiimide hydrochloride in 0.1 M MES buffer. Samples were incubated at room temperature for 2 h using a rotary shaker, and washed three times with PBS. Samples were then soaked in 500 μL of 1/10 fluorescein-conjugated streptavidin (GeneTex) diluted with PBS, incubated at room temperature for 1 h in the dark, and washed three times with PBS. Fluorescent and reflected light images were acquired using CLSM, as described above.

### TEM analysis

Microbialite samples fixed with 2.5% glutaraldehyde/creek water were post-fixed with 1.5% OsO_4_ in 100 mM cacodylate buffer, pH 7.4, for 90 min, and embedded in an epoxy resin (EPOK 812, Oken). 800 nm thin sections were first obtained using an ultramicrotome (Ultracut E, Reichert-Jung), and transmitted and cross-polarized light images were acquired after toluidine blue staining. 70−80 nm thin sections were obtained, stained with 2% uranyl acetate and lead citrate, and montage images were acquired using TEM (JEM-1400, Jeol) operated at an accelerating voltage of 80 kV. To analyze the relationship between the exopolymers and minerals, thin-foil sections were prepared from resin embedded samples using a focused-ion beam (FIB) apparatus (SMI4050, Hitachi), and observed with TEM (JEM-ARM200F, Jeol) operated at an accelerating voltage of 200 kV, as described previously^41^. The elemental composition was examined using energy-dispersive X-ray spectroscopy (EDS) installed with the TEM, and combined elemental maps were generated by scanning TEM (STEM) with Analysis Station 3.8 software.

### STXM analysis

STXM-based NEXAFS analysis of carbon (1s) and calcium (2p) were performed using the BL13A beamline at KEK-PF (Tsukuba, Japan), of which the general experimental setup has been described previously^42^. Model compounds for Ca NEXAFS measurements were obtained from Nichika Inc. (calcite and aragonite) and Wako Pure Chemical Ltd. (calcium oxide), and the ground powders were deposited onto a carbon-coated copper grid (Cu 200 mesh, Jeol). For sample analysis, the surface of fresh stromatolite was scraped with a sterile knife, suspended in distilled water, dropped onto a carbon-coated copper grid, and air dried at room temperature. Sample preparation and STXM analysis were conducted within 24 h and 56 h after sampling, respectively. Compositional images were generated with the RGB composite mode of aXis 2000 software^43^ using images of specific absorption edges for protein (288.2 eV), acidic polysaccharide (288.6 eV)^44–49^, and calcium (352.6 eV)^13^. In addition, the same thin-foil sections used for TEM analysis were also analyzed using STXM.

## Electronic supplementary material

Supplementary Information
