# Supplementary material for: Cyanobacterial exopolymer properties differentiate microbial carbonate fabrics
Source: Sci Rep. 2017 Sep 18;7:11805. doi: 10.1038/s41598-017-12303-9 (PMC5603507; doi:10.1038/s41598-017-12303-9)
Supplement: Supplementary file 1 — Supplementary Information [file 41598_2017_12303_MOESM1_ESM.pdf]

Cyanobacterial exopolymer properties differentiate microbial carbonate fabrics

– Supplementary Information –

Fumito Shiraishi[1], Yusaku Hanzawa[1], Tomoyo Okumura[2,3], Naotaka Tomioka[4], Yu Kodama[5], Hiroki Suga[1], Yoshio Takahashi[3] & Akihiro Kano[3]

[1]Department of Earth and Planetary Systems Science, Graduate School of Science, Hiroshima University, Hiroshima 739-8526, Japan.
[2]Department of Subsurface Geobiological Analysis and Research (D-SUGAR), Japan Agency for Marine-Earth Science and Technology (JAMSTEC), Yokosuka 237-0061, Japan.
[3]Department of Earth and Planetary Science, The University of Tokyo, Tokyo 113-0033, Japan.
[4]Kochi Institute for Core Sample Research, JAMSTEC, Kochi 783-8502, Japan.
[5]Marine Works Japan Ltd., Yokosuka 237-0063, Japan.

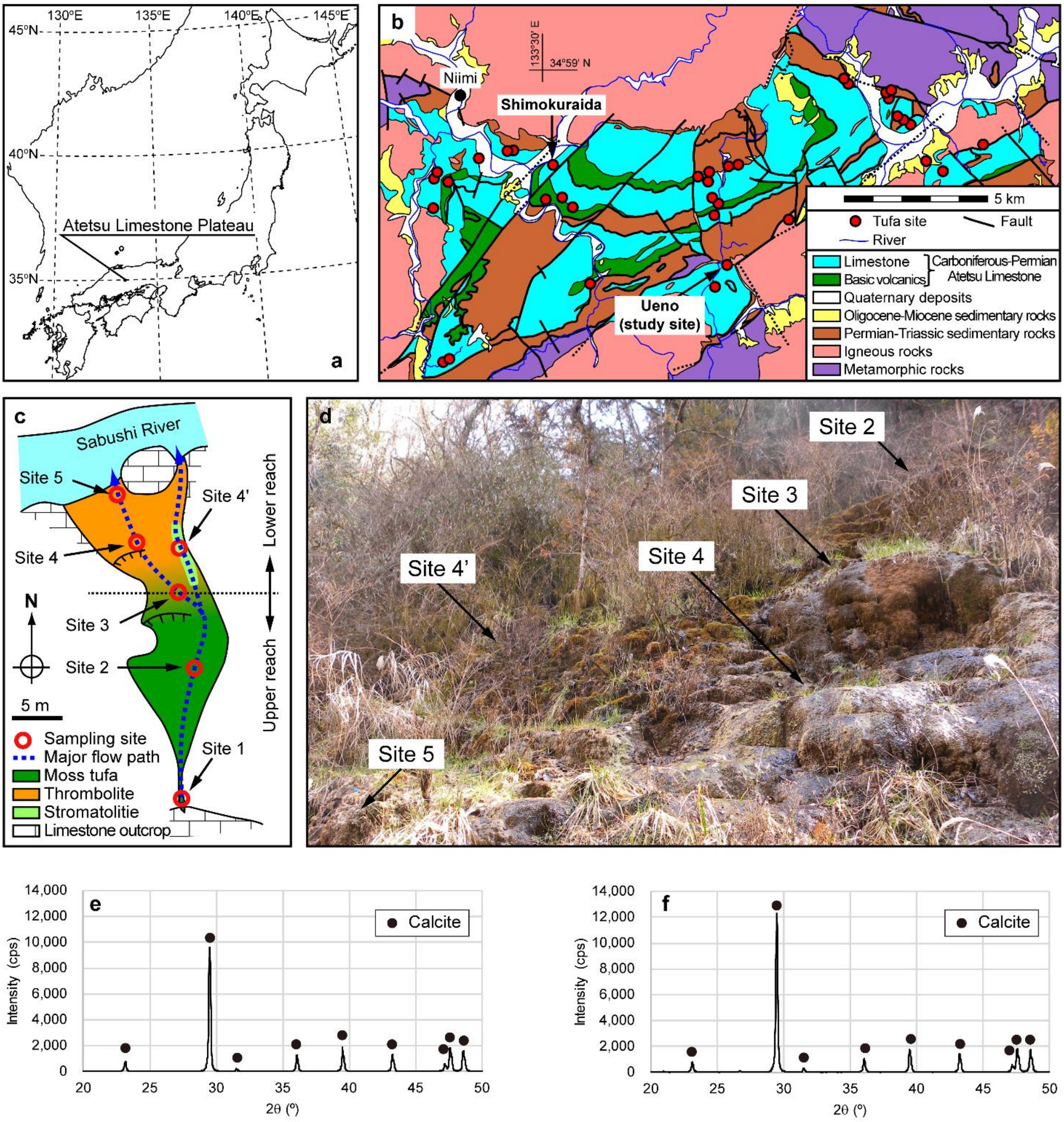

g

	pH	Water temp.	Alkalinity	Na^+	K^+	Ca^{2+}	Mg^{2+}	Cl^-	NO_3^-	SO_4^{2-}	DIC	Ω	pCO_2		Mg/Ca	$\delta^{13}C_{water}$	$\delta^{18}O_{water}$	$\delta^{13}C_{tufa}$	$\delta^{18}O_{tufa}$
		(ºC)	(meq L^{-1})	(mmol L^{-1})	(mmol L^{-1})	(mmol L^{-1})	(mmol L^{-1})	(mmol L^{-1})	(mmol L^{-1})	(mmol L^{-1})	(mmol L^{-1})		(matm)	(PAL)		(‰ SMOW)	(‰ SMOW)	(‰ PDB)	(‰ PDB)
Site 1	6.99	13.6	4.51	0.11	0.01	2.54	0.02	0.09	0.01	0.04	5.62	0.7	23.4	58.6	0.008	-14.2	-9.0		
Site 2	7.61	13.7	4.44	0.11	0.01	2.46	0.02	0.09	0.01	0.04	4.68	2.6	5.5	13.7	0.008	-13.0	-8.9	-11.8	-7.4
Site 3	7.89	13.8	3.29	0.11	0.02	1.90	0.02	0.10	0.01	0.04	3.37	3.0	2.1	5.3	0.011	-10.8	-9.0	-10.9	-7.5
Site 4	7.98	13.2	3.27	0.11	0.01	1.84	0.02	0.09	0.01	0.04	3.32	3.5	1.7	4.3	0.011	-10.6	-8.8	-10.1	-7.5
Site 4'	8.19	13.5	3.85	0.12	0.01	2.16	0.02	0.09	0.01	0.04	3.84	7.4	1.2	3.1	0.010	-11.6	-9.0	-11.5	-7.3
Site 5	7.86	13.9	3.19	0.11	0.01	1.83	0.02	0.09	0.01	0.04	3.27	2.6	2.2	5.6	0.011	-10.5	-8.7	-9.8	-7.7

Supplementary Figure S1. Field setting of the Ueno tufa site. The maps were drawn by F.S. using Adobe Illustrator CS6 (Version number: 16.0.0, URL link: http://www.adobe.com/). (**a**) Location of

Atetsu Limestone Plateau, southwestern Japan. (**b**) Geological map of Atetsu Limestone Plateau, showing 36 tufa sites reported to date[45]. Locations of Ueno and well-studied Shimokuraida[9,46–48] tufa sites are indicated. (**c**) Detailed map of the Ueno tufa site showing the distribution pattern of three different deposit types (moss tufa, thrombolite, and stromatolite) and six sampling sites. (**d**) Field view of Ueno tufa site, in which the locations of five sampling sites among six are indicated. (**e, f**) XRD patterns of (**e**) stromatolite and (**f**) thrombolite, which indicate that the major mineralogy is calcite for both microbialites. (**g**) Physicochemical characteristics of creek water. Although features common to general tufa-depositing creeks[8,9,47,49] were observed, certain differences were recognized between western and eastern flow paths. In the eastern flow path (Site 4'), water flow faster in a narrow channel; stronger water agitation stimulated CO_2 degassing to cause larger increases of pH and the saturation state of calcite (Ω), while decreases of Ca^{2+} concentration and alkalinity were relatively suppressed due to a shorter time for calcite precipitation. $p CO_2$ was well below the threshold for the CO_2 concentrating mechanism (10 times the present atmospheric level[29]; PAL) both at stromatolite-depositing (Site 4') and thrombolite-depositing sites (Sites 4 and 5).

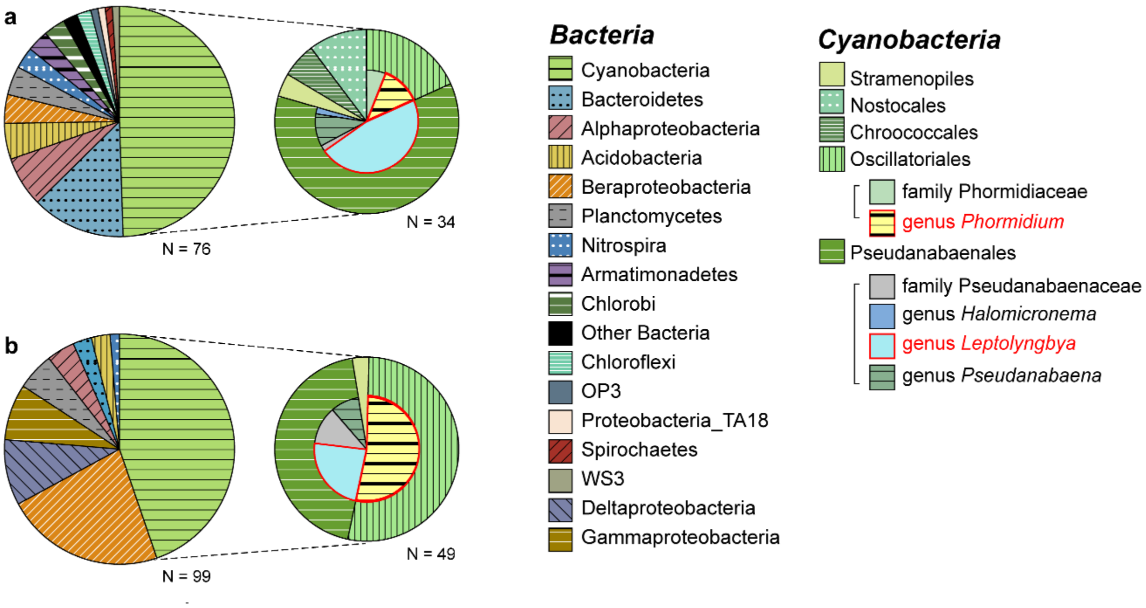

c

Phylotypes	Stromatolite (%)	Thrombolite (%)
Cyanobacteria	45	49
Bacteroidetes	3	13
Alphaproteobacteria	4	7
Acidobacteria	3	5
Beraproteobacteria	22	4
Planctomycetes	5	4
Nitrospira	1	3
Armatimonadetes	0	3
Chlorobi	0	3
Other Bacteria	0	2
Chloroflexi	0	2
Candidate division OP3	0	1
Proteobacteria TA18	0	1
Spirochaetes	0	1
Candidate division WS3	0	1
Deltaproteobacteria	9	0
Gammaproteobacteria	8	0

d

Phylotypes			Stromatolite	Thrombolite
Order	Family	Genus	(%)	(%)
Stramenopiles			1	2
Nostocales			0	5
Chroococcales			0	3
Oscillatoriales			24	9
	Phormidiaceae		(0)	(3)
		Phormidium	(24)	(6)
Pseudanabaenales			20	30
	Pseudanabaenaceae		(5)	(1)
		Halomicronema	(0)	(1)
		Leptolyngbya	(11)	(23)
		Pseudanabaena	(4)	(5)

Supplementary Figure S2. Bacterial community compositions in Ueno microbialites. (**a, b**) Phylotype composition of bacterial phylum (left) and cyanobacterial order (right) in (**a**) the stromatolite and (**b**) the thrombolite. The number of taxa was higher in the thrombolite than in the stromatolite. Approximately half of the total clones belonged to the phylum *Cyanobacteria* for both the stromatolite and the thrombolite, although their constituents were different; genus *Phormidium* was dominant in the stromatolite with a subordinate proportion of genus *Leptolyngbya*, while the trend was opposite in the thrombolite (highlighted by red frames). (**c, d**) Tables showing the breakdowns of (**c**) bacterial phylum and (**d**) cyanobacterial order/family/genus. The parentheses numbers indicate the content ratios of each order-level phylotypes.

a

pH	Water temp.	Alkalinity	Na^{+}	K^{+}	Ca^{2+}	Mg^{2+}	Cl^{-}	NO_3^{-}	SO_4^{2-}	DIC	Ω	pCO_2	
	(ºC)	(meq L^{-1})	(mmol L^{-1})	(mmol L^{-1})	(mmol L^{-1})	(mmol L^{-1})	(mmol L^{-1})	(mmol L^{-1})	(mmol L^{-1})	(mmol L^{-1})		(matm)	(PAL)
7.99	15.0	4.6	0.14	0.04	2.80	0.02	0.12	0.01	0.04	4.65	7.4	2.3	5.9

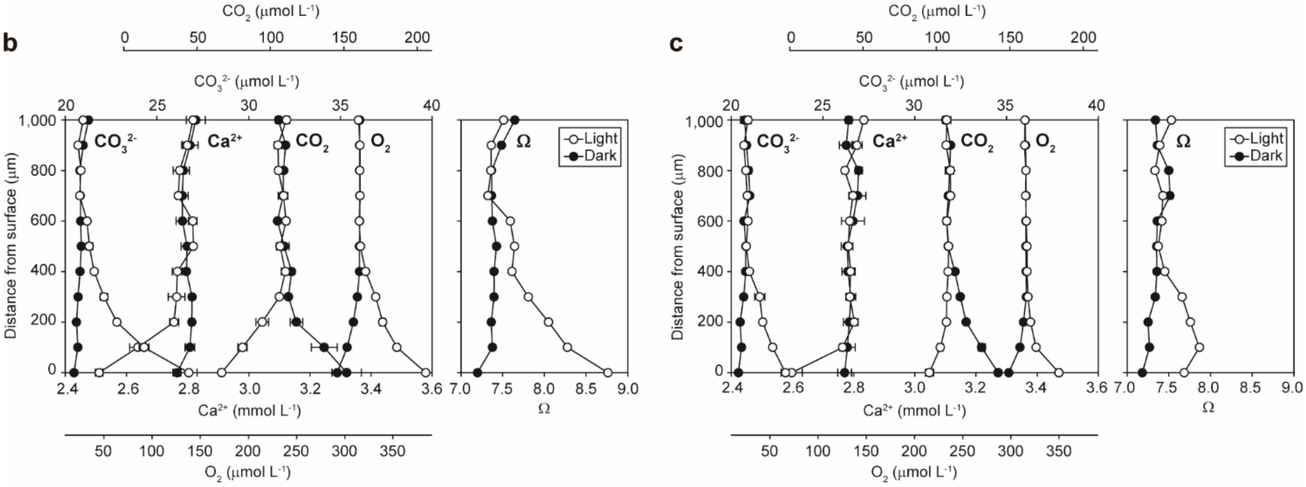

d

Sample	Condition	O_2	CO_2	CO_3^{2-}	Ca^{2+}	Organic matter production / calcification ratio
Stromatolite	Light	6.34 (±2.29) × 10^{-7}	-1.73 (±0.80) × 10^{-7}	1.26 (±0.70) × 10^{-8}	-1.04 (±0.53) × 10^{-6}	0.36
	Dark	-2.57 (±0.90) × 10^{-7}	3.38 (±0.84) × 10^{-7}	n.d.	n.d.	
Thrombolite	Light	7.65 (±2.44) × 10^{-7}	-1.33 (±0.64) × 10^{-7}	5.67 (±1.92) × 10^{-9}	-6.76 (±3.21) × 10^{-7}	0.93
	Dark	-1.33 (±0.50) × 10^{-7}	1.93 (±0.34) × 10^{-7}	n.d.	n.d.	

Supplementary Figure S3. Microelectrode data. (**a**) Physicochemistry of water used for microelectrode measurements. (**b, c**) Microelectrode profiles measured at the surface of (**b**) stromatolite and (**c**) thrombolite. Open and closed circles indicate light (ca. 500 μE m^{-2} s^{-1}) and dark profiles, respectively. Error bars represent the standard deviation (s.d.) of the measured values (n = 3). It is noted that each profile was not measured exactly at the same point, which occasionally causes some difficulties for the precise estimation of Ω. (**d**) Fluxes of dissolved components (mol m^{-2} s^{-1}) at the microbialite surface calculated from microelectrode profiles. Positive and negative values indicate the fluxes from and toward the microbialite surface, respectively. Bracketed numbers represent the s.d. of repeated measurements at different points (n = 3−5). The organic matter production/calcification ratio was calculated from the net O_2 flux divided by the net Ca^{2+} flux. The ratio of thrombolite was greater than that of stromatolite, which is opposite to that suggested in a previous study[9].

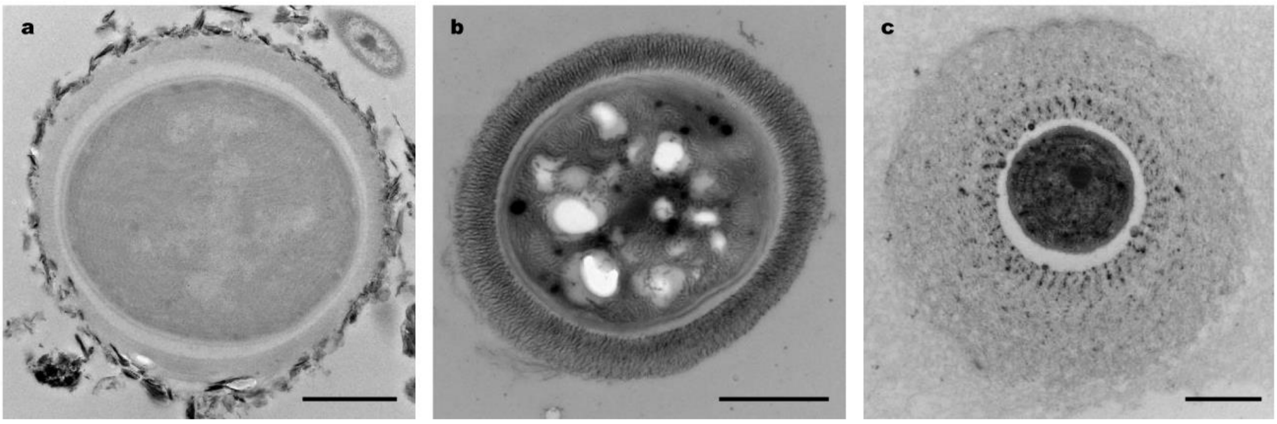

Supplementary Figure S4. TEM images of dominant cyanobacteria. Cross-sections of (**a**) *Phormidium* sp., (**b**) *Leptolyngbya* sp., and (**c**) *Coelosphaeriopsis* sp. (Scale bars: 1 μm.)

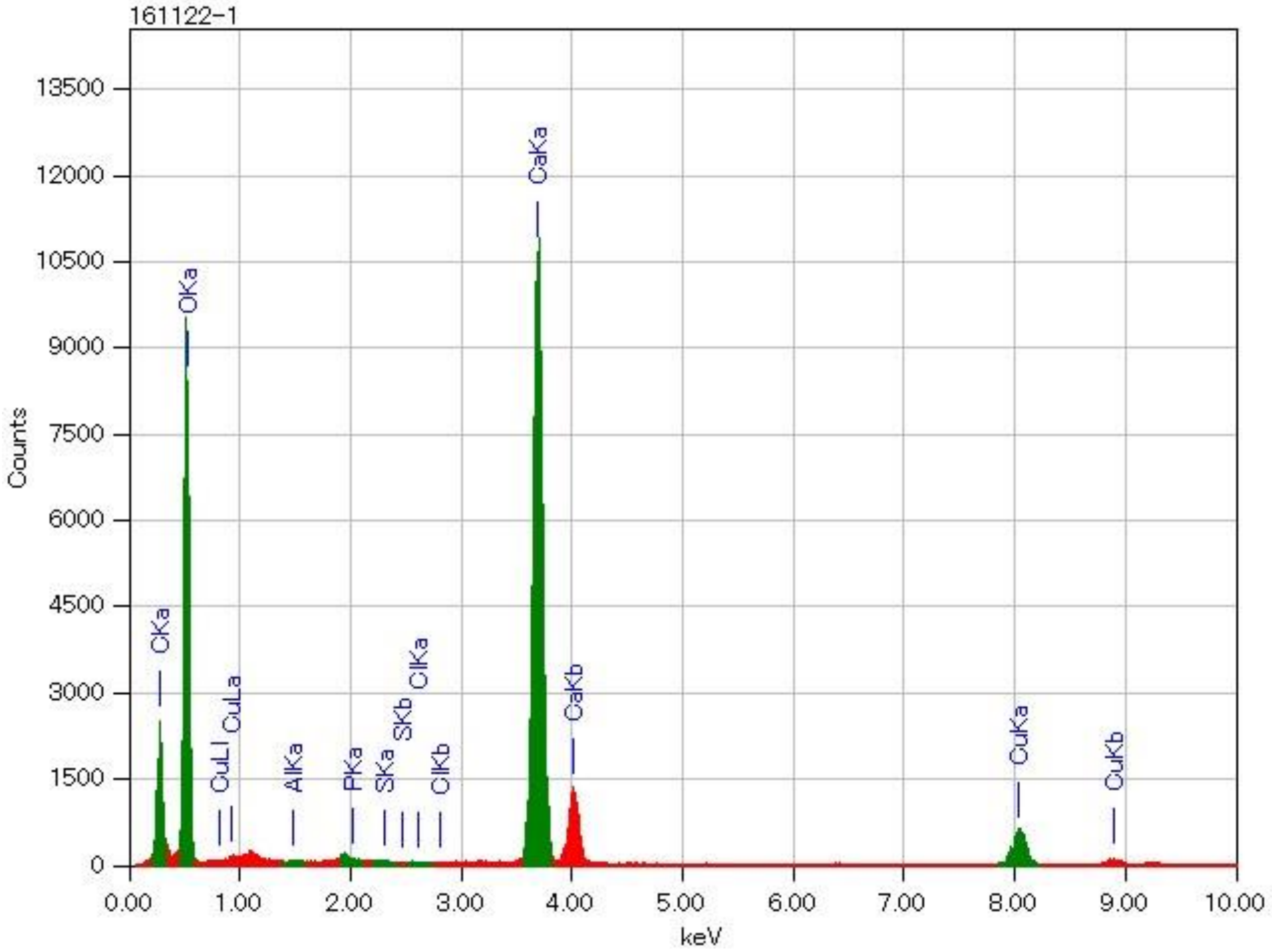

Supplementary Figure S5. An EDS spectrum obtained from spot 5 shown in Fig. 1f. Mg was undetectable. Cu peaks represent the influence from a Cu grid stabilizing the thin-foil section.

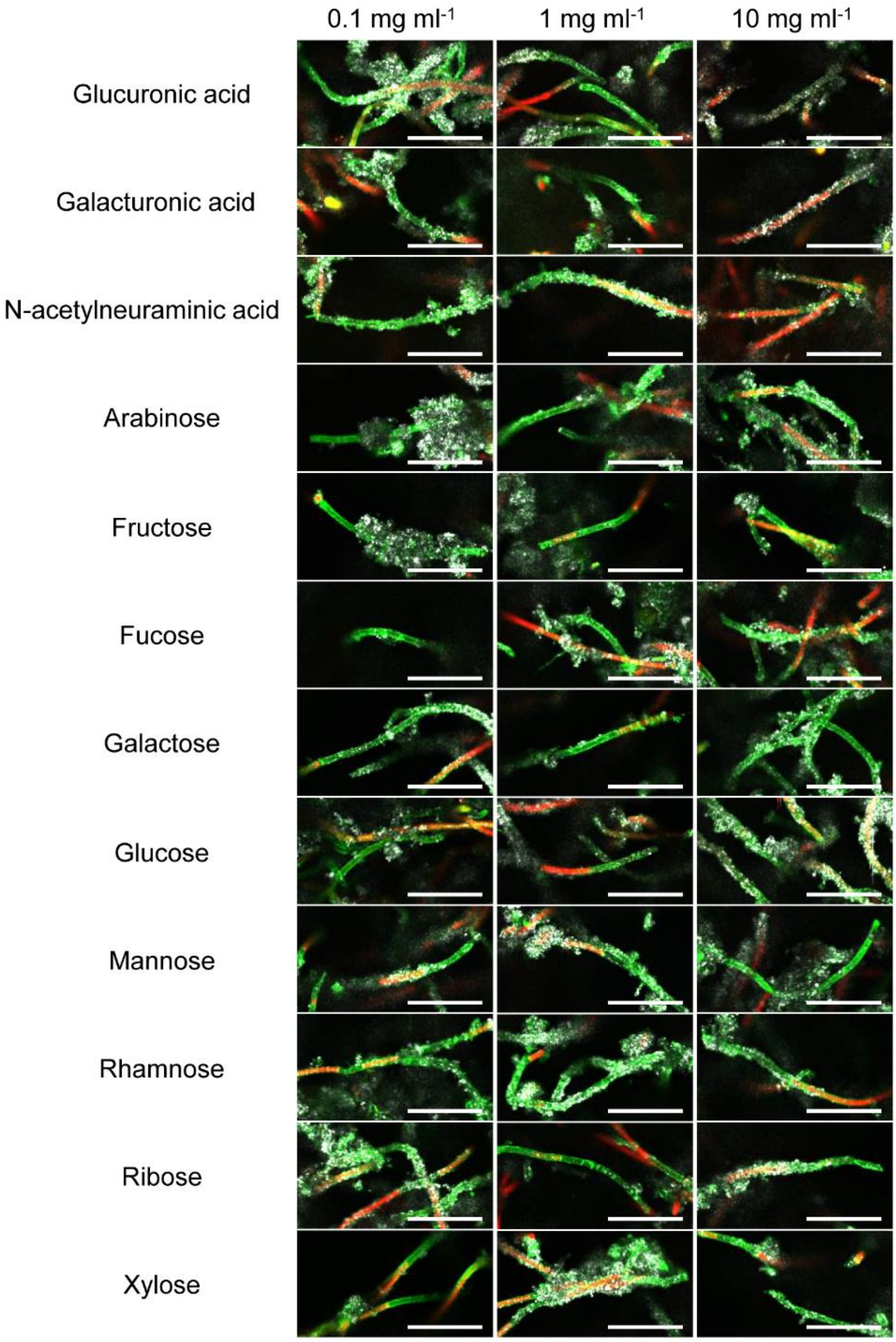

Supplementary Figure S6. Lectin blocking assay for LPA lectin. Among 12 competing sugars tested, only acidic sugars (glucuronic acid, galacturonic acid, and N-acetylneuraminic acid) recognizably reduced the intensity of LBA at the highest concentration. This result indicates that LPA lectin has binding specificity to these acidic sugars. (Scale bars: 50 μm.)

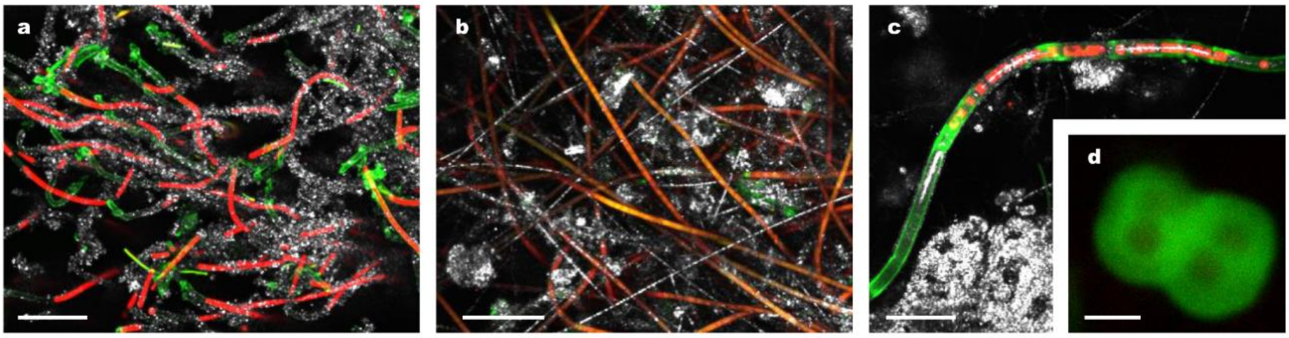

Supplementary Figure S7. Fluorescence labeling of carboxyl groups. (**a**–**d**) CLSM images of microbialite surfaces, in which phototrophs, carboxyl groups, and minerals are presented by yellow-red autofluorescence, green fluorescence, and white reflected light, respectively. (**a**) Stromatolite surface inhabited by *Phormidium* sp., and thrombolite surface inhabited by (**b**) *Leptolyngbya* sp., (**c**) *Scytonema* sp., and (**d**) *Coelosphaeriopsis* sp. The results are comparable to the LBA results (Figs. 1c, 2c), which supports the interpretations together with the lectin blocking assay. (Scale bars: **a**–**c**, 50 μm; **d**, 2 μm.)
